# Non-invasive electrical stimulation for sleep disturbances in adults: protocol for an evidence−mapping umbrella review of systematic reviews and meta−analyses with subgroup analysis by intervention type and population

**DOI:** 10.3389/fpsyt.2026.1857226

**Published:** 2026-05-28

**Authors:** Jingyi Zhu, Yitong Lin, Yuanyuan Chen, Baixiao Zhao

**Affiliations:** 1 School of Acupuncture-Moxibustion and Tuina, Beijing University of Chinese Medicine, Beijing, China; 2 Dongzhimen Hospital of Beijing University of Chinese Medicine, Beijing, China

**Keywords:** insomnia, meta-analyses, non-invasive electrical stimulation, sleep disturbances, systematic reviews, umbrella review

## Abstract

**Background:**

Sleep disturbances affect 10%–30% of adults worldwide. Non−invasive electrical stimulation (e.g., transcranial electrical stimulation) has emerged as a promising non−pharmacological intervention. Although numerous systematic reviews and meta−analyses have been published, they vary considerably in methodological quality, populations, intervention types, and conclusions. No umbrella review has yet synthesised the evidence across different modalities and populations. This evidence mapping umbrella review aims to systematically chart the existing systematic reviews, assess methodological quality, quantify overlap, and describe evidence patterns across diverse modalities and populations.

**Methods:**

Following JBI guidelines, we will search PubMed, Embase, Cochrane Database of Systematic Reviews, Web of Science, PsycINFO, and Scopus (inception to April 2026), restricted to English. Grey literature will be searched via PROSPERO, ClinicalTrials.gov, Google Scholar (first 200 records), and reference list screening (snowballing). We will include systematic reviews and meta−analyses of randomised controlled trials evaluating any non−invasive electrical stimulation for sleep outcomes. Two reviewers will independently screen, extract data, and assess methodological quality using AMSTAR 2. Primary study overlap will be quantified by the Corrected Covered Area. Where feasible, we will calculate 95% prediction intervals, perform Egger’s regression tests, and conduct excess significance tests using review-level summary estimates. Subgroup analyses will be stratified by intervention and population type. Sensitivity analyses will exclude: (1) reviews with critically low AMSTAR 2 ratings, (2) preprints, (3) reviews at high risk of reporting bias, and (4) studies where sleep is not the primary outcome. The primary outcome is subjective sleep quality; total sleep time is a key secondary outcome. Evidence will be graded using the Fusar−Poli classification, with GRADE for key outcomes.

**Discussion:**

This umbrella review will provide the highest level of evidence synthesis, identifying modalities with more consistent or higher certainty evidence and highlighting areas where evidence remains uncertain. Limitations include restriction to English (which may disproportionately impact modalities such as TEAS), expected heterogeneity, and possible insufficient data for some subgroup analyses. All amendments have been documented in PROSPERO (CRD420261357590).

**Clinical Trial Registration:**

https://www.crd.york.ac.uk/prospero/, identifier CRD420261357590.

## 1 Introduction

Sleep disturbances, including primary insomnia and sleep problems comorbid with physical or psychiatric conditions, affect approximately 10% to 30% of adults worldwide (1, 2). These conditions impose a substantial health burden, such as reduced quality of life, lower work productivity, increased cardiovascular risk, and a higher prevalence of depression and anxiety (3). Despite this high prevalence and burden, insomnia remains insufficiently recognised and undertreated in many regions (4).

Conventional treatments consist of pharmacotherapy (e.g., benzodiazepines, Z−drugs, melatonin receptor agonists) (5) and cognitive−behavioural therapy for insomnia (CBT−I) (6). Pharmacological options have notable adverse effects, including daytime sedation, dependence, and cognitive impairment, and are not recommended for long−term use (7, 8). CBT−I is a first−line non−pharmacological treatment with sustained efficacy, yet its widespread adoption is limited by a shortage of trained therapists, high costs, and poor patient adherence (9–11). Therefore, safe, effective, accessible and low−cost alternatives are urgently needed.

Non−invasive electrical stimulation (NIES) techniques have emerged as a promising class of interventions for sleep improvement (12–14). Common modalities include transcranial electrical stimulation, transcutaneous electrical acupoint stimulation, transcutaneous auricular vagus nerve stimulation, transcutaneous electrical nerve stimulation, and other peripheral nerve stimulation techniques. These methods deliver low−intensity currents through surface electrodes placed on the scalp, auricle, acupoints or peripheral nerves, modulating neural activity without surgery or implanted devices. NIES is non−invasive, portable, relatively low−cost, and potentially self−administered at home, making it attractive for long−term sleep management (15).

Numerous systematic reviews and meta−analyses have evaluated NIES for sleep, but the existing evidence has significant limitations. The study populations vary considerably: some reviews focus on primary insomnia (16), whereas others include comorbid sleep disturbances associated with psychiatric disorders, neurological conditions, chronic pain or postoperative recovery (17, 18). Intervention types, stimulation parameters and comparator conditions also differ substantially across reviews, making direct comparisons difficult (19, 20). Moreover, findings are inconsistent. Some reviews report significant improvements, while others show no benefit or high heterogeneity (16). These limitations highlight the need for an umbrella review to synthesise and critically appraise the existing evidence.

To date, no umbrella review has systematically synthesised the totality of evidence on NIES for sleep improvement across different modalities and populations. An umbrella review is one of the highest levels of evidence synthesis. It aggregates findings from multiple systematic reviews and meta−analyses, assesses their methodological quality, quantifies primary study overlap, tests the robustness of effect estimates, and grades evidence strength using standardised criteria (21).

This evidence mapping umbrella review aims to systematically identify, map, and critically appraise all published systematic reviews and meta-analyses evaluating non-invasive electrical stimulation for sleep outcomes in adults. The primary purpose is to chart the distribution of effect estimates across intervention and population subgroups, identify evidence clusters and gaps, and provide a clear evidence hierarchy. This structure is intended to inform future research priorities and contribute to a more precise understanding of which modalities have consistent or higher certainty evidence and which remain uncertain, rather than to issue immediate clinical recommendations.

## 2 Methods and analysis

This umbrella review will be designed and conducted in strict accordance with the Joanna Briggs Institute (JBI) methodology for umbrella reviews (22), which defines an umbrella review as a type of evidence synthesis that systematically integrates existing systematic reviews and meta−analyses, aiming to provide the highest level of evidence summary for clinical decision−making. Regarding reporting standards, we will follow the PRIOR statement (Preferred Reporting Items for Overviews of Reviews) (23), a reporting guideline specifically developed for overviews of reviews (including umbrella reviews), which comprises 27 items covering the title, abstract, introduction, methods, results, discussion, and other sections. For specific items not covered by PRIOR, the PRISMA 2020 statement will be consulted as a supplement (24). The present protocol has been written in accordance with the PRISMA−P framework and appropriately adapted to the characteristics of an umbrella review. This protocol has been registered with PROSPERO (registration number: CRD420261357590), and any amendments will be documented in PROSPERO with the date and reasons for the change.

### 2.1 Inclusion and exclusion criteria

According to the PICOS (Population, Intervention, Comparator, Outcome, Study design) framework, the inclusion criteria will be organized into the following five components.

#### 2.1.1 Population

##### 2.1.1.1 Inclusion criteria

Adults aged ≥18 years, with no upper age limit. Three population types will be included: (1) primary insomnia disorder, diagnosed according to standardized criteria (DSM-5, ICSD-3, or DSM-IV) (25); (2) comorbid sleep disturbances, i.e., sleep complaints occurring in the context of other physical or psychiatric conditions, including postoperative pain, depression, anxiety, Parkinson’s disease, chronic pain (e.g., fibromyalgia, osteoarthritis, low back pain), and other conditions (e.g., cancer, cardiovascular disease, stroke, multiple sclerosis); (3) Non−clinical poor sleepers, i.e., no formal diagnosis but reporting poor sleep quality (e.g., Pittsburgh Sleep Quality Index [PSQI] >5) or self-reported sleep difficulties.

##### 2.1.1.2 Exclusion criteria

Studies focusing exclusively on sleep-disordered breathing (e.g., obstructive sleep apnea) where insomnia is not the primary outcome; studies that treat pregnant or postpartum women as a distinct population. Pregnant and postpartum women are excluded because the safety and efficacy of non-invasive electrical stimulation in these populations involve distinct physiological considerations that require a separate focused review. However, where a broader review (e.g., covering all adults with a given condition) reports separate subgroup data for these populations, these data will be extracted and summarised narratively.

#### 2.1.2 Intervention

Non-invasive electrical stimulation (NIES), including but not limited to: central electrical stimulation (transcranial electrical stimulation [tES]: tDCS, tACS, tRNS, CES; transcutaneous spinal cord stimulation [tSCS]; transcutaneous spinal electroanalgesia [TSE]); peripheral nerve stimulation (transcutaneous electrical acupoint stimulation [TEAS], transcutaneous auricular vagus nerve stimulation [taVNS], transcutaneous electrical nerve stimulation [TENS], trigeminal nerve stimulation [TNS], median nerve stimulation [MNS], remote electrical neuromodulation [REN], temporal interference stimulation [TIS], percutaneous electrical nerve field stimulation [PENFS]); microcurrent and low-intensity stimulation (microcurrent electrical therapy [MET], frequency-specific microcurrent [FSM]); galvanic vestibular stimulation (GVS), electrical vestibular nerve stimulation (VeNS); and other forms (neuromuscular electrical stimulation [NMES], functional electrical stimulation [FES], interferential current therapy [IFC]). No restrictions will be placed on stimulation parameters (current intensity, frequency, pulse width, electrode placement, number of sessions). Electroacupuncture requiring needle insertion is considered an invasive technique and will be excluded. Non-invasive electrical stimulation of acupoints via surface electrodes (e.g., TEAS) is included.

#### 2.1.3 Comparator

Sham stimulation (placebo), waitlist control, usual care, no intervention, or active comparator. Active comparators will be documented but not included in the primary analyses.

#### 2.1.4 Outcome

Primary outcome: Subjective sleep quality, measured by validated self-report instruments such as the Pittsburgh Sleep Quality Index (PSQI) or the Insomnia Severity Index (ISI). These instruments are the most consistently reported outcomes across systematic reviews of non-invasive electrical stimulation and are recommended as core sleep endpoints in clinical trials.

Secondary outcomes: (1) Total sleep time (TST), measured by polysomnography (PSG), actigraphy, or validated sleep diaries. Self-reported TST from sleep diaries is acceptable as a secondary measure and will be analysed separately from objectively measured TST where both are available. (2) Sleep onset latency (SOL). (3) Sleep efficiency (SE). (4) Wake after sleep onset (WASO). (5) Adverse events and tolerability.

Timing: Immediately post-intervention and at follow-up (≥1 month, if available).

#### 2.1.5 Study design

##### 2.1.5.1 Inclusion criteria

(1) Systematic reviews or meta-analyses in which the included primary studies are randomised controlled trials (RCTs); (2) if a systematic review includes both RCTs and non-randomised studies, it will be included only if the RCT data can be extracted separately; otherwise it will be excluded; (3) Cochrane systematic reviews will be included.

##### 2.1.5.2 Exclusion criteria

Animal studies; study types including narrative reviews, scoping reviews, commentaries, primary studies (e.g., RCTs, cohort studies), conference abstracts without full text, and protocols without results. Umbrella reviews (overviews of reviews) or other tertiary evidence syntheses will be excluded from the main synthesis; they will be used solely for reference list screening (snowballing) and as background sources in the Discussion.

### 2.2 Information sources and search strategy

We will search databases from their inception to 30 April 2026, with no lower date limit, and only English language publications will be included. The comprehensive search strategy will cover the following databases: PubMed, Embase, Cochrane Database of Systematic Reviews (CDSR), Web of Science, PsycINFO, and Scopus. To minimise publication bias and identify ongoing or unpublished research, we will also search the PROSPERO register of systematic reviews and ClinicalTrials.gov for relevant protocols and registered trials. In addition, a supplementary search will be conducted using Google Scholar (the first 200 records will be screened to balance recall and workload), and we will manually screen the reference lists of all included systematic reviews (snowballing) to identify any additional eligible reviews not captured by the electronic searches. Umbrella reviews and overviews of reviews identified through these searches will not be included in the main synthesis; instead, their reference lists will be screened to identify additional eligible systematic reviews of primary studies. **Table 1** presents the search strategy developed for the PubMed database. The search query combines Medical Subject Headings (MeSH) and free-text keywords using Boolean operators (AND, OR). The search strategies for other databases will be adapted according to their respective syntax. Complete search strategies for all databases are provided in Supplementary File 1.

**Table 1:** PubMed search strategy.

Number	Search terms
#1	Electric Stimulation Therapy[Mesh] OR “Transcutaneous Electric Nerve Stimulation”[Mesh] OR “Vagus Nerve Stimulation”[Mesh] OR “Spinal Cord Stimulation”[Mesh]
#2	transcranial electric* stimulation[tiab] OR “tES”[tiab] OR “transcranial direct current stimulation”[tiab] OR “tDCS”[tiab] OR “transcranial alternating current stimulation”[tiab] OR “tACS”[tiab] OR “transcranial random noise stimulation”[tiab] OR “tRNS”[tiab] OR “cranial electrotherapy stimulation”[tiab] OR “CES”[tiab] OR “electrosleep”[tiab] OR “transcutaneous electric* acupoint stimulation”[tiab] OR “TEAS”[tiab] OR “transcutaneous auricular vagus nerve stimulation”[tiab] OR “taVNS”[tiab] OR “auricular vagus nerve stimulation”[tiab] OR “transcutaneous electric* nerve stimulation”[tiab] OR “TENS”[tiab] OR “peripheral nerve stimulation”[tiab] OR “transcutaneous spinal cord stimulation”[tiab] OR “tSCS”[tiab] OR “microcurrent electrical therapy”[tiab] OR “MET”[tiab] OR “non-invasive electric* stimulation”[tiab] OR “noninvasive electric* stimulation”[tiab] OR “interferential current therapy”[tiab] OR “IFC”[tiab] OR “frequency-specific microcurrent”[tiab] OR “FSM”[tiab] OR “percutaneous electrical nerve field stimulation”[tiab] OR “PENFS”[tiab] OR “remote electrical neuromodulation”[tiab] OR “REN”[tiab] OR “trigeminal nerve stimulation”[tiab] OR “TNS”[tiab] OR “median nerve stimulation”[tiab] OR “MNS”[tiab] OR “temporal interference”[tiab] OR “TIS”[tiab] OR “vestibular nerve stimulation”[tiab] OR “galvanic vestibular stimulation”[tiab] OR “GVS”[tiab] OR “VeNS”[tiab]
#3	Sleep Initiation and Maintenance Disorders[Mesh] OR “Sleep Wake Disorders”[Mesh]
#4	sleep[tiab] OR “insomnia”[tiab] OR “sleep quality”[tiab] OR “sleep disorder”[tiab] OR “sleep disturbance”[tiab] OR “sleep maintenance”[tiab] OR “Pittsburgh Sleep Quality Index”[tiab] OR “PSQI”[tiab] OR “Insomnia Severity Index”[tiab] OR “ISI”[tiab] OR “total sleep time”[tiab] OR “TST”[tiab] OR “sleep onset latency”[tiab] OR “SOL”[tiab] OR “sleep efficiency”[tiab]
#5	systematic review[pt] OR “meta-analysis”[pt] OR “systematic review”[tiab] OR “meta-analysis”[tiab] OR “umbrella review”[tiab] OR “overview of reviews”[tiab] OR “review of reviews”[tiab] OR “evidence synthesis”[tiab] OR “network meta-analysis”[tiab]
#6	#1 OR #2
#7	#3 OR #4
#8	#6 AND #7 AND #5

### 2.3 Study selection and data extraction

#### 2.3.1 Study selection

The study selection process will be conducted by two independent reviewers (JYZ and YTL) using the systematic review management platform Rayyan:

1. Title and abstract screening: articles that fail to meet the inclusion criteria will be excluded.
2. Full-text screening: the full text of potentially eligible reviews will be obtained and assessed against the eligibility criteria. All reviews meeting the inclusion criteria will proceed to data extraction and methodological quality appraisal regardless of their AMSTAR 2 rating. No review will be excluded at this stage solely on the basis of low quality. Umbrella reviews encountered during screening will be flagged and set aside; their reference lists will be examined for potentially eligible systematic reviews not yet captured, but they will not proceed to data extraction or be included in the evidence synthesis.
3. Disagreements will be resolved through discussion, and if unresolved, by consulting a third reviewer (YYC).

The study selection process will be detailed in a PRISMA flow chart.

#### 2.3.2 Data extraction

Data will be extracted into a standardised Excel form, including: basic information (first author, year, journal, PROSPERO registration status); intervention characteristics (type: tES, TEAS, taVNS, TENS, tSCS, MET, NMES, IFC, etc., specific technique, stimulation parameters if reported); study characteristics (number of primary studies included, total sample size (intervention/control)); population characteristics (population type: primary insomnia, comorbid sleep disturbances with specification of the comorbid condition: postoperative pain, depression, anxiety, Parkinson’s disease, chronic pain, others, diagnostic criteria, mean age); outcomes and effect estimates (outcome name, outcome type (primary/secondary), effect size type (SMD/MD/RR), pooled effect size with 95% confidence interval, P value, heterogeneity I², model type (fixed/random)); publication bias (method used: Egger’s test or funnel plot, Egger’s P value, funnel plot symmetry); quality assessment (AMSTAR 2 overall rating and assessment of the seven critical domains); overlap assessment (list of primary studies included in each systematic review for CCA calculation); and other information (country of corresponding author, funding source, conflicts of interest). For other eligible studies with missing or incomplete outcome data, we will contact the corresponding authors by email to request the necessary information. The process will be conducted independently by two researchers (JYZ and YTL), who will cross-check the extracted data to ensure completeness and accuracy. Any discrepancies will be resolved through consultation with a third party (YYC).

### 2.4 Methodological quality assessment

The methodological quality of the included systematic reviews will be assessed using the AMSTAR 2 tool (26). Two reviewers will independently perform the assessment, and disagreements will be resolved through discussion. The seven critical domains to be evaluated are: Item 2 – whether the protocol was pre-registered; Item 4 – whether the search strategy was comprehensive; Item 7 – whether a list of excluded studies with justifications was provided; Item 9 – whether the risk of bias assessment of primary studies was appropriate; Item 11 – whether the synthesis methods were appropriate; Item 13 – whether heterogeneity was considered and explained; and Item 15 – whether publication bias was assessed. An overall confidence rating (high, moderate, low, or critically low) will be assigned according to AMSTAR 2 criteria. The process will be conducted independently by two researchers (JYZ and YTL), who will cross-check the extracted data to ensure completeness and accuracy. Any disagreements will be resolved through consultation with a third party (YYC).

### 2.5 Data synthesis and summary of evidence

#### 2.5.1 Narrative synthesis

A descriptive summary will be provided, organised by intervention type (e.g., tES, TEAS, taVNS, TENS, tSCS, MET, NMES, IFC) and population type (primary insomnia; comorbid sleep disturbances further stratified by comorbid condition: postoperative pain, depression, anxiety, Parkinson’s disease, chronic pain, others; and Non−clinical poor sleepers).

#### 2.5.2 Quantitative synthesis (where feasible)

For each eligible meta-analysis, we will extract the pooled effect estimate (with its 95% confidence interval) as reported in the most recent or highest-quality systematic review. Prediction intervals will be calculated using the random-effects model based on these review-level summary estimates. Egger’s regression test and the excess significance test (Ioannidis and Trikalinos method) will also be conducted at the meta-analysis level using the reported effect sizes and variances of the individual primary studies as presented in each review. We will not re-extract or re-analyse raw data from primary trials unless a critical outcome has not been meta-analysed in any included review and the review provides sufficient individual-study data to permit a new synthesis. The “≥10 studies” threshold refers to the number of primary studies within a single meta-analysis, not to the number of systematic reviews per outcome. For the excess significance test, the plausible true effect size will be assumed equal to the pooled estimate from the most recent and highest-quality meta-analysis for each outcome, or a standardised mean difference (SMD) of 0.2 if no reliable prior estimate is available. All statistical analyses will be performed using R (version 4.x; R Foundation for Statistical Computing, Vienna, Austria) with the meta, metafor, and umbrella packages. RevMan (The Cochrane Collaboration) will be used for certain descriptive summaries and risk-of-bias figures where applicable.

For outcomes with sufficient data (≥10 studies for statistical tests), the following analyses will be performed:

① Prediction intervals: 95% prediction intervals (PI) will be calculated to assess the uncertainty of effect estimates in future studies. If the 95% PI crosses the null, the robustness of the evidence will be considered low.
② Small-study effects: Egger’s regression test will be used to detect publication bias; a two-tailed P < 0.10 will be considered indicative of small-study effects.
③ Excess significance test: The method of Ioannidis and Trikalinos will be used to test whether the observed number of statistically significant studies (P < 0.05) exceeds the expected number. A significant excess (P < 0.05) may suggest selective reporting or publication bias.

All statistical analyses will be performed using R (version 4.x; R Foundation for Statistical Computing, Vienna, Austria) with the meta, metafor, and umbrella packages. RevMan (The Cochrane Collaboration) will be used for certain descriptive summaries and risk of bias figures where applicable.

#### 2.5.3 Subgroup analyses

If sufficient data are available (≥3 systematic reviews per subgroup), the following stratified analyses will be performed:

① Intervention type: tES, TEAS, taVNS, TENS, tSCS, MET, NMES, IFC, others.
② Population type: primary insomnia, comorbid sleep disturbances, Non−clinical poor sleepers.

#### 2.5.4 Sensitivity analyses

Sensitivity analyses will be conducted by:

① Excluding systematic reviews with a critically low AMSTAR 2 rating;
② Excluding preprints (if included) or grey literature retrieved from Google Scholar, etc.;
③ Excluding systematic reviews at high risk of reporting bias;
④ Including only studies where sleep is the primary outcome (to test the robustness of the findings against the inclusion of studies where sleep is a secondary outcome).

#### 2.5.5 Overlap assessment

The degree of primary study overlap across the included systematic reviews will be quantified using the Corrected Covered Area (CCA):

CCA = (N - r)/(r × c - r).

where N = total number of primary study inclusions (counting duplicates), r = number of unique primary studies, and c = number of systematic reviews. Overlap will be classified as slight (0–5%), moderate (6–10%), high (11–15%), or very high (>15%).

If the corrected covered area (CCA) indicates high (11–15%) or very high (>15%) overlap, we will graphically map primary study overlap using a matrix and narratively describe the degree of redundancy. For each intervention-outcome pair with substantial overlap, we will present the evidence primarily from the most recent, highest-quality (AMSTAR 2 rating), and largest (by number of included trials) systematic review.

The corrected covered area matrix and related overlap visualisations will be generated using a custom R script.

#### 2.5.6 Grading of evidence

This umbrella review will adopt a dual approach to evidence grading:

Fusar-Poli classification (21) (primary method): According to the umbrella review evidence grading criteria proposed by Fusar-Poli and Radua, each outcome will be classified into one of five levels:

- Class I (convincing evidence): P < 10^-6^, total sample size >1000, no small-study effects (Egger’s P ≥ 0.10), 95% prediction interval does not cross the null, no excess significance bias, high or moderate AMSTAR 2 quality.
- Class II (highly suggestive evidence): P < 10^-6^, total sample size >1000, with minor methodological limitations.
- Class III (suggestive evidence): P < 0.001.
- Class IV (weak evidence): P < 0.05, but with evidence of small-study effects, high heterogeneity (I² > 50%), or low/critically low AMSTAR 2 quality.
- NS (non-significant): P ≥ 0.05.

For key outcomes (e.g., Pittsburgh Sleep Quality Index), we will additionally apply the GRADE (Grading of Recommendations Assessment, Development and Evaluation) framework to rate the certainty of evidence as high, moderate, low, or very low. This assessment will be based on the standard GRADE domains: risk of bias, inconsistency, indirectness, imprecision, and publication bias. Because this umbrella review is designed to map and grade the existing evidence rather than formulate clinical practice guidelines, we will not issue strength of recommendations.

## 3 Discussion

This umbrella review is the first to systematically synthesise the evidence on different non−invasive electrical stimulation techniques for improving sleep in adults. We have minimised publication bias by comprehensively searching multiple English databases (PubMed, Embase, Cochrane Library, Web of Science, Epistemonikos) and supplementing with searches of PROSPERO, ClinicalTrials.gov, Google Scholar, and the reference lists of included studies. Methodologically, the quality of included systematic reviews will be assessed using AMSTAR 2, the degree of primary study overlap will be quantified using the Corrected Covered Area (CCA), and subgroup analyses by intervention type and population type are pre−specified. Statistically, 95% prediction intervals, Egger’s test, and excess significance tests will be calculated to examine the robustness of effect estimates. Evidence strength will first be graded using the Fusar−Poli classification, with GRADE applied to key outcomes.

This study has several limitations. Restricting to English−language publications may introduce language bias and omit important systematic reviews in other languages, and this restriction may have a disproportionate impact on evidence for modalities such as TEAS, where a substantial body of studies is published in Chinese. Future updates should therefore consider including Chinese databases with appropriate quality safeguards. Moreover, the methodological quality of the original systematic reviews may vary; however, sensitivity analyses excluding critically low−quality reviews will test the robustness of the main conclusions. Considerable differences in stimulation parameters and sleep outcome measures across systematic reviews may lead to high heterogeneity, and for subgroups with insufficient data (fewer than three reviews), only a narrative summary will be possible. In addition, the grey literature search using Google Scholar will screen only the first 200 records, potentially missing a few relevant documents, and new literature published after the search date will not be included.

Nevertheless, this umbrella review is expected to provide a comprehensive map of the existing evidence, describing which techniques (e.g., taVNS, TEAS) are supported by more consistent or higher certainty evidence and which currently lack robust support. The findings will inform future research priorities and more focused systematic reviews; however, definitive clinical conclusions cannot be drawn until the full review is completed and its limitations are fully assessed. It will also systematically map existing research gaps and propose directions for future research: more high−quality randomised controlled trials with standardised stimulation protocols, adequate sample sizes, and long−term follow−up (≥6 months) are needed; systematic reviews should adhere to AMSTAR 2 standards and the PRIOR reporting guideline; and understudied populations such as older adults and specific comorbid groups should receive more attention. According to the timeline, literature screening, data extraction, and quality assessment will be completed between May and October 2026, and data analysis and drafting of the first manuscript will be completed by December 2026. The conclusions of this protocol reflect the planned methodology; the actual strength of evidence and any potential clinical implications will be determined only after the umbrella review has been completed and all findings have been critically appraised.

The protocol was prospectively registered in PROSPERO (CRD420261357590) to ensure transparency and reproducibility. All amendments made during the revision process have been documented in the registry to maintain consistency between the published protocol and the final review.
